# What Is the Remedy?

**Published:** 1888-10

**Authors:** 


### WHAT IS THE REMEDY?

Some time ago we gave symptoms, asking for the corresponding remedies. Our subscribers have requested us to give them some more exercises of this kind, so we now give a few more.

1. In a recumbent posture the mental faculties and memory are perfect, but on every attempt to move, vertigo?

2. Headache internally, with sensation when turning of something loose in head, diagonally across top?

3. Sensation of stiffness around the eyes and in the eyelids; eye symptoms worse in the evening and in the open air?

4. Nausea; everything becomes black before the eyes, with pressure in the throat, incarcerated flatus, oppression of breathing, and rheumatic pains in the limbs?

5. Pressure in the pit of stomach, like a marble; worse when sitting erect, with sensation as if something would be pressed off below the pit of the stomach?

6. Sudden pains in paroxysms, across abdomen above umbilicus, from lower border of liver downward toward the left, then ceasing in right side; worse from motion, better when sitting up?

7. Palpitation up to the throat after going to bed; trembling all over; worse lying on right side; better lying on the back; anxiety?

8. Shooting, stabbing pain from the heart through to the left scapula, causing violent beating of the heart?

9. Pains worse sitting bent, yet feeling as if to do so was necessary, but relieved by sitting or standing upright? [Gastralgia.]

10. Pain occurring at irregular times, continuing for no definite period, coming suddenly or gradually and leaving as uncertainly? [Neuralgia.]

